# Ethnomedicinal and phytochemical review of Pakistani medicinal plants used as antibacterial agents against *Escherichia coli*

**DOI:** 10.1186/s12941-014-0040-6

**Published:** 2014-08-19

**Authors:** Muhammad Adnan, Roqaia Bibi, Sakina Mussarat, Akash Tariq, Zabta Khan Shinwari

**Affiliations:** 1 Department of Botany, Kohat University of Science and Technology, Kohat 26000, Pakistan; 2 Department of Biotechnology, Quaid-i-Azam University Islamabad, Islamabad, 44000, Pakistan

**Keywords:** Medicinal plants, Biological screening, Ethnomedicines, Phytochemistry, Bacteria

## Abstract

Medicinal plants have always been part of human culture and have the potential to cure different diseases caused by microorganisms. In Pakistan, biologists are mainly focusing on plants’ antimicrobial activities against *Escherichia coli* due to its increasing resistance to antibiotics. In total, extracts from 34 ethnomedicinally valuable Pakistani plants were reported for *in-vitro* anti-*E. coli* activities. Mostly methanolic extracts of medicinal plants were used in different studies, which have shown comparatively higher inhibitory activities against *E. coli* than n-hexane and aqueous extracts. It has been found that increasing concentration (mg/ml) of methanolic extract can significantly increase (p < 0.01) anti-*E. coli* activities. Not all medicinal plants are extracted in solvents others than above, which should also be tested against *E. coli*. Moreover, medicinal plant species must be fully explored phytochemically, which may lead to the development of new drugs.

## Introduction

Therapeutic properties of medicinal plants are well recognized at global level [1]. As an estimate, over 50% of modern clinical drugs have natural products’ origin [2]. World Health Organization has emphasized on the use of traditional medicines and reported about 80% of population from developing countries relies on medicinal plants for their primary health care [3],[4]. It is believed that more than 8,000 plants species in South Asia carries medicinal properties, of which 1000 exists in Pakistan [5]. Local people use these medicinal plants for the treatment of various ailments through their indigenous knowledge [6]. However, due to modernization, traditional medicines are only practiced in remote rural areas [7],[8].

In Pakistan, pathogenic bacteria are causing serious infectious diseases like gastro-intestinal, pneumonia, pulmonary and skin related. A number of Pakistani medicinal plants have been tested for their antimicrobial activities [9]. These plants contain different phytochemicals such as alkaloids, glycosides, saponins, resins, oleoresins, sesquiterpene lactones and oils (essential and fixed). Other compounds like furanocoumarins, hydroxycoumarins, napthoquinones, acylphloroglucinols and sterones have also been isolated from these species. It was identified that 74% of the 119 plant derived drugs were discovered as a result of isolation of active substances from medicinal plants [10].

*Escherichia coli* are gram negative bacteria, and mainly responsible for urinary tract and gastro-intestinal infections in human [11]. They are the best and most studied free-living microorganisms [12],[13]. Some strains of *E. coli* live as harmless commensalism in animals’ intestines while others causes serious diseases. These strains included enteropathogenic, enterohemorrhagic, enteroinvasive, enterotoxigenic, and enteroaggregative [14]. The enterohemorrhagic *E. coli* strain (EHEC) O157:H7 was first recognized as a gastro-intestinal pathogen in 1982 and became a world-wide public health problem [15]. However, most of the diseases caused by these bacteria are being treated locally using medicinal plants. Different methods like biological screening, isolation of compounds and clinical trials have been used to find out the efficacy of medicinal plants against microorganisms causing a particular disease [16],[17].

Emergence of multiple drug resistant bacterial strains due to indiscriminate use of antibiotics has generated a keen interest in the discovery of effective plants derived drugs [18]. *E. coli* are showing increased resistance to different antibiotics like amoxicillin and trimethoprim [19],[20]. Hence, searching of alternative and effective medicines from plants against such resistant bacteria has become an important concern all over the world [21]. Antibiotics on one side became ineffective to bacterial strains but also costly for the poor communities of developing world [22],[23]. Furthermore, the antibiotics may be associated with adverse effects including hypersensitivity and immune suppression [24]. Therefore, this review was designed with the aim to (i) compile the available fragmented literature on anti-*E. coli* effect of Pakistani medicinal plants, and (ii) suggest measures on newer and safer herbal drugs for the diseases caused by the *E. coli*. Furthermore, this review will provide knowledge on ethnomedicines and phytochemistry of those Pakistani medicinal plants having anti-*E. coli* potential. Above all, this review will provide baseline information for chemists, pharmacists and pharmacologists to carry out in-depth *in-vitro* and *in-vivo* activities for the development of novel drugs against *E. coli* with low cost and less side effects on living system.

## Methodology

### Literature selection

Online literature on antibacterial activities of Pakistani medicinal plants against *E. coli* was searched and gathered using online bibliographic databases including Google Scholar, ISI Web of Knowledge and Science Direct Navigator, as well as some libraries sources. An extensive number of published and unpublished articles and reports were found on Pakistani medicinal plants extracted with different solvents (methanol, ethanol, ethyl acetate, n-hexane, chloroform etc.) for theirs *in-vitro* biological screening. In total, 112 plants were found tested for their *in-vitro* anti-*E. coli* in Pakistan. However, this review consisted of 34 plants, on which sufficient information were available regarding extracts’ concentrations (mg/ml) necessary for maintaining uniformity in the data. This study is the combination of anti-*E. coli* activities, ethnomedicinal properties and phytochemistry of reported medicinal plants that were collected from the available literature.

### Extraction techniques used in Pakistan

Extraction is the process of separation of active metabolites of medicinal plants using different solvents through standard procedures. Common techniques used in Pakistan for extraction process are Maceration, Infusion, Percolation, Decoction and Soxhlet [25],[26]. Maceration is the most proffered technique, in which powdered plant-drug is kept in a container with solvent for a defined period with frequent stirring until soluble matter is dissolved [27].

### Data organization and statistical analysis

**Figure 1:**
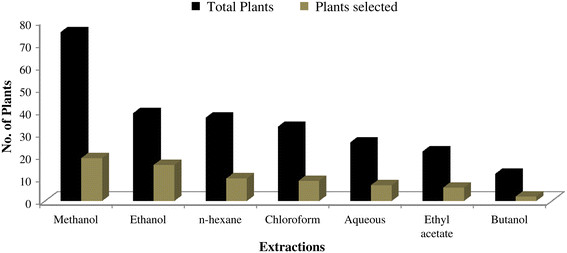
**Pakistani medicinal plants extracted with different solvents.** Plants selected indicate those that are selected out of total species for this review.

**Figure 2:**
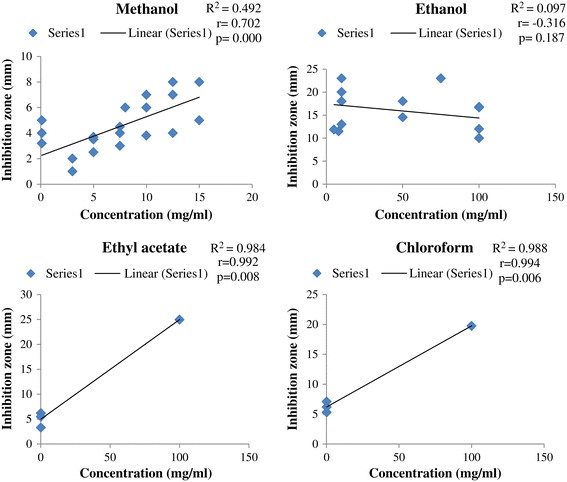
**Pearson correlations between medicinal plant extracts’ concentrations (mg/ml) and inhibition zones of** ***E. coli*** **(mm).**

**Figure 3:**
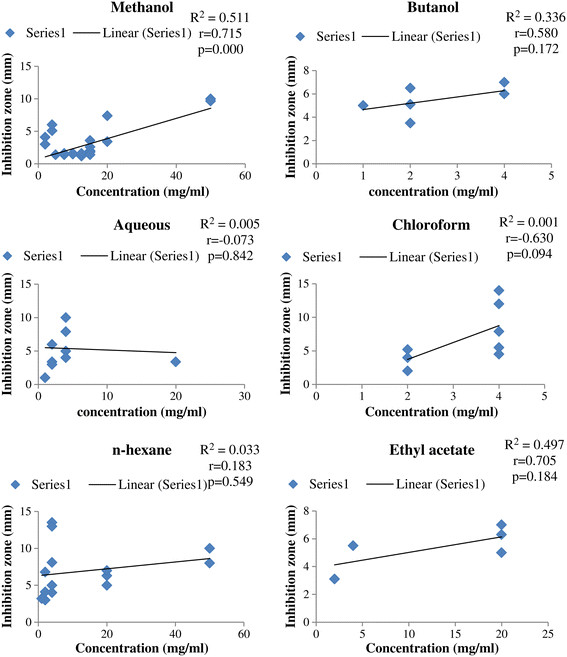
**Pearson correlations of different concentration of medicinal plants extracts dissolved in DMSO solvent (mg/ml) and inhibition zones of** ***E. coli*** **(mm).**

**Figure 4:**
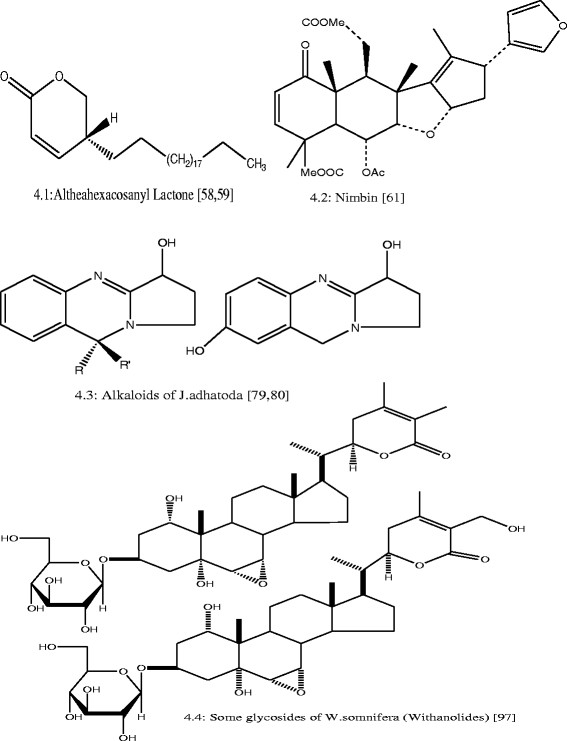
Structure of chemical compounds isolated from plants having antibacterial activities.

Data was organized and tabulated using Microsoft Excel 2007 and Word 2007. First table was designed on the *in-vitro* anti-*E. coli* activities of Pakistani medicinal plants. This table consisted of data on the concentrations of plant extracts (uniformed to mg/ml) and their anti-*E. coli* zone of inhibition (uniformed to mm). Second table composed of ethnomedicinal properties and phytochemistry of reported medicinal plants. Figure 1 depicts total number of medicinal plants used against *E. coli* in Pakistan, which justifies the criteria of species’ selection for this review. Pearson correlation was applied using SPPS between plant extracts’ concentrations and anti-*E. coli* zone of inhibition (Figures 2 and 3). Furthermore, Figure 4 was developed in Chemdraw, which illustrates active phytochemical of selected medicinal plants having anti-*E. coli* activities.

## Discussion

### Medicinal plants extracted with methanol and ethanol

Methanol and ethanol are organic compounds used for the extractions of different medicinal plants. These solvents are mostly preferred throughout the world for extraction process [28]. Present review showed that majority of plant species (19) were extracted using methanol solvent (Figure 1). The higher use of methanol might be associated with its higher antibacterial activities in comparison to other solvents. Statistically plant extracts’ of methanolic concentrations (mg/ml) in DMSO have significantly increased the anti-*E. coli* inhibitory activities (Figures 2 and 3).

Ethanolic extracts of certain plants also showed excellent inhibitory activities. Anti-*E. coli* inhibitory activities of methanol and ethanol might be related to their polar nature, due to which these solvents can easily degrade the cell wall of medicinal plants and helps in releasing polyphenols from cells. Ncube *et al.*[27] mentioned that polyphenols are best in their antibacterial activities. Polyphenols are organic in nature, which can be obtained through methanolic and ethanolic extractions [16].

#### Withania somnifera

*W. somnifera* is widely used as traditional medicine in remote areas of Pakistan for various ailments (Table 1). Mahmood *et al.*[29] described that methanolic extract of *W. somnifera* at different concentrations 15, 10, 5 and 3 mg/ml showed 8, 7, 3.7 and 1 mm inhibition against *E. coli*, respectively. Leaves extract of *W. somnifera* showed 18 mm inhibition against *E. coli* at 10 mg/ml concentration [30] (Table 2). These strong antibacterial activities of *W. somnifera* might be due to the presence of Withanolides (Figure 4.3), which have been isolated from the leaves [31] (Table 1).

**Table 1:** Ethnobotany and phytochemistry of Pakistani medicinal plants

**Plant Species/Family names**	**Part used**	**Extract**	**Phytoconstituents**	**Ethnobotany**	**Mode of preparation**	**Route of admin.**	**References**
*Althaea officinalis* Linn Malvaceae	Seed, root, leaves, flower	Methanol	n-hexacos-2-enyl-1,5-olide (altheahexacosanyl lactone), 2β-hydroxycalamene (altheacalamene) and 5,6-dihydroxycoumarin-5-dodecanoate-6β-D-glucopyranoside (altheacoumarin glucoside), lauric acid, β-sitosterol and lanosterol. Dihydrokaempferol 4′-O-glucoside, Tiliroside, Hypolaetin 8-0-gentiobioside	Expectorant, demulcent, burns, snake bite, asthma, bronchitis pneumonia, rheumatism, kidney and bladder problems	Decoctions of the plant, especially of the root, are very useful for intestinal problems. Seeds, leaves and flowers are boiled in wine or milk and taken to relieve diseases like chest, coughs, bronchitis and whooping-cough.	Oral, dermal	[32]–[34]
Azadirachta indica *Adr. Juss.* Meliaceae	Leaves	Ethanol	Azadirachtin	Antiseptic, digestive and gastric problems, skin diseases, stomach flatulence	Decoction of leaves is taken for digestive and gastric problems. Fresh leaves are boiled in water and tied on wounds. Leaves are dried, crushed and powder is mixed with small quantity of water and taken for the remedy of freckles on face and increase appetite by lessening stomach flatulence and killing worms.	Oral, dermal	[35]–[37]
*Calendula arvensis* L. Compositae	Leaves	Ethanol	28-O-β-D-glucopyranoside-3-β-O-(O-β-D-galactopyranosyl (1 → 3)-β-D-glucopyranoside.	Hepatitis and spleen enlargement control	Decoction of leaves is used as required.	Oral	[35],[38]
			3-β-O-(O-β-D-galactopyranosyl (1 → 3)-β-D-glucopyranoside.				
*Calotropis procera* Ait. f., Hort. Solanaceae	Stem, leaves	Methanol, aqueous	alkaloids, flavonoids, tannins, steroids, triterpenoids, saponins	Expectorant, anthelmentic, cholera, asthma, earache, pyorrhea, gastro-intestinal diseases	Stem latex is used in earache and asthma. Infusion of leaves used for stomach problems.	Oral, dermal	[35],[39]
*Cannabis sativa* L. Cannabaceae	Leaves	Ethanol	Anhydrocannabisativine	Sedative, anodyne, narcotic	Whole plant extract is effective cure of livestock dysentery. *Cannabis* is also used for the treatment of number of condition including AIDS, multiple sclerosis and thermotherapy induced nausea. Its decoction is used for the treatment of the cancer, neuro protection, fever and high blood pressure. It cause hallucination when drunk in excessive quantity.	Oral, dermal	[40]–[42]
			Cannabisativine				
			cannabinoids				
			N-(p-hydroxy-β-phenylethyl)-p-hdroxy-(trans)-cinnamide				
*Carum copticum* L. Apiaceae		Methanol, ethanol, n-hexane, acetone		Appetizers, kidney stone, digestion and whooping cough	Seeds are taken with little salt for gas trouble as stomach tonic.	Oral	[36],[43]
*Cichorium Intybus* L. Asteraceae	Root	Methanol	[lup-12,20 (29)-dien-3β-ol-3β-L-arabinofuranosyl-2′ -hexadecanoate]	Abdominal pain, diarrhea	The whole plant is used for carminative purposes.	Oral	[34],[40],[44]
			[lup-12,20 (29)-dien-3β-olyl hexadecanoate]				
			[4β-(pent-2-enylolactone)-hexatriacontane]				
*Cinnamomum zeylanicum* Blume. Lauracaeae	Kohat	n-hexane	carbohydrates, alkaloids, tannins, steroids, tannins, flavonoids, glycosides	Toothache and sore gums, carminative, stimulant, anti-microbial, anti-fungal	Bark is boiled in water and makes tea and sipped.	Oral	[45]
*Cistanche tubulosa* (Schenk) R. Wight. Orobanchaceae	Stem	Methanol	Glycosides, monoterpenes				[46]
*Datura innoxia* Mill. Solanaceae	Seed	Methanol	Daturadiol, daaturaolone, 3β,6β-dihydroxyoleane-12-ene, 3-oxo-6β-hydroxyoleane-12-ene	anti-inflammatory, laxative antispasmodic, sedative, malaria	Smoke of the plant is inhaled to cure asthma. Fruits are used in malaria. Fruit is used to reveal cardiac pains and distress. Leaves are used in earache. The juice of the fruit is applied to the scalp for curing dandruff and falling hairs.	Dermal	[34],[35],[47]
*Delonix regia* L. Leguminosae	Stem, bark, leaves	Ethanol, methanol	L-Azetidine-2-carboxylic acid, lupeol, epilupeol, b-sitosterol, stigmasterol and p- methoxybenzaldehyde alkaloids, tannnins, triterpenoids, steroids, glycosides, flavonoids, so-flaflavones, flavones, anthocyanine, coumarines, lignins, vitamin-A, vitamin-E, vitamin-C.ß-Amyrin, hesperitin	Abdominal pains, bronchitis and pneumonia	Root decoction is used for abdominal pains and in the treatment of scorpion bite. The leaves extract is used as anti-inflammatory. The herb is also used in bronchitis and pneumonia in infants. It is used as a carminative.	Oral	[48]–[50]
*Dodonaea viscosa* L. Capparidaceae	Leaves, aerial parts	Ethanol	Tannins, saponins, flavanoids and terpenoids	Astringent, anti rheumatic, swelling cutaneous, skeletal and gastro-intestinal diseases and burns	Grind the leaves and add small amount of water (Infusion) to make fine paste for dermal use.	Oral, dermal	[40],[51]
*Eucalyptus camaldulensis* Dehnh. Myrtaceae	Leaves	Ethanol	Ellagitannins, flavonoids, phloroglucinol derivatives and galloyl esters.	Flu and cold	Five to ten leaves boiled in water and decoction is taken for flu twice a day.	Oral	[36],[52]
*Ficus carica* L. Moraceae	Leaves, fruit	Ethanol	Steroids, triterpenoids, cumarines, flavanoids and glycoside	Respiratory, gastro intestinal, urinary and cutaneous diseases, demulcent, laxative, antiseptic, constipation, flatulence, measles, dysentery, bladder problems and verrucas	Burn the leaves and the ash is sprayed on the wounds dermally. Decoction is used for intestinal problems. Fresh fruit used in anemia and constipation. Latex obtained from the stem and leaves for checking bleeding.	Oral, dermal	[34],[53]
*Glycyrrhiza glabra* L. Leguminosae	Root	Methanol	Glycyrrhizin	Respiratory illness, cough	Dried root are crushed and powder taken orally.	Oral	[54]
*Hyssopus officinalis* L. Lamiaceae	Leaves	Methanol	a-Glucosidase inhibitors. quercetin 7-O-β-D-apiofuranosyl-(1 → 2)-β-D-xylopyranoside and quercetin 7-O-β-D-apiofuranosyl-(1 → 2)-β-D-xylopyranoside3′-O-β-D-glucopyranoside	asthma, cough, bronchitis, fever, trauma, rheumatism			[55],[56]
*Justicia adhatoda* L. Acanthaceae	Leaves	Methanol	Alkaloids	Diuretic, jaundice, antispasmodic cough, asthma, bronchitis, tuberculosis, rheumatism, gastro-intestinal, diarrhea, dysentery, antimicrobial	Grind the leaves and mix it with honey. The paste is used dermally around the swelling. Decoction is used for respiratory diseases and diarrhea.	Oral, dermal	[57]–[59]
*Malva neglecta* Wall. Malvaceae		Methanol		Purgative			[40]
*Malva sylvestris* L. Malvaceae	Leaves, root, flower	Methanol	(2-methyl-3-methoxy-5,6-dihydroxy-1,4-naphthoquinone)				[60]
*Mentha longifolia* L. Lamiaceae	Leaves	Ethanol	Longifone, (longiside-A and -B) and flavanone-glycoside (longitin) tricetin 7-O-methylether 3′-O-glucoside 5′-O-rhamnoside, tricetin 3′-O-glucoside 5′-O-rhamnoside and tricetin 3′-O-rhamnosyl- 1 → 4 –rhamnoside	Carminative, diarrhea, dysentery and stomachache	The dried plant is use for the treatment of diarrhea and its “*chatenii*” is good tonic for improvement of stomach. The tea of leaf with lemon extract is common household tonic to cure cold, flu, respiratory disorders.	Oral	[40],[61],[62]
*Olea europaea*. L Oleaceae	Leaves	Ethanol	Oleuropein, Hydroxytyrosol, Leteoline-7-glucoside				[63],[64]
*Otostegia limbata* (Benth.) Boiss Labiatae	Leaves, root	Ethanol, methanol	5-hydroxy-2-(4-hydroxyphenyl)-4-oxo-7-[(α-L-rhamnopyranosyl) oxy]-4H-chromen-3-yl β-D-glucopyranosyl-(1 → 2)-[β-D-glucopyranosyl-(1 → 4)]-[6-O-[(2E)-3-(4-hydroxyphenyl) prop-2-enoyl]-β-D-glucopyranosyl-(1 → 3)]-α-L-rhamnopyranoside, 5-hydroxy-2-(4-hydroxyphenyl)-4-oxo-7-[(α-L-rhamnopyranosyl) oxy]-4H-chromen-3-yl [6-O-[(2E)-3-(4-hydroxyphenyl) prop-2-enoyl]-β-D-glucopyranosyl-(1 → 2)]-[β-D-glucopyranosyl-(1 → 4)]-[6-O-[(2E)-3-(4-hydroxyphenyl) prop-2-enoyl]-β-D-glucopyranosyl-(1 → 3)]-α-L-rhamnopyranoside	Wounds, gum diseases, dental, cutaneous diseases		Oral	[35],[65]
*Paeonia emodi* Wall. ex Royle Paeoniaceae	Root, flower, leaves	Methanol	Monoterpenes, monoterpene glycosides, triterpenoids, flavonoids, phenols and tannins	Backache, epilipsy, convulsions, uterine diseases, vomiting, cholera, whooping cough, diarrhea	The leaves of *Ruta graveolens*, *Paeonia emodi* root, are grounded together and sieved through a cloth. *Mamordica charantia* and water are mixed together and added to sufuf formed.	Oral, dermal	[34],[66]–[68]
*Phyllanths emblica* L. Euphorbiaceae	Fruit, leaves	Methanol	kaempferol-3-O-α-L-(6″-methyl)-rhamnopyranoside, kaempferol-3-O-α-L-(6″-ethyl)-rhamnopyranoside 5-hydroxymethylfurfural Qeurcetin, gallicacid	Carminative, stomachic, diuretic, laxative cooling effect, asthma, bronchitis, scurvy, cardiac, tuberculosis, diabetes, gonorrhea, rheumatism, jaundice, dysentery, diarrhea	Dried fruits are grind and taken with water against dysentery and diarrhea	Oral	[34],[69],[70]
*Ricinus communis* L. Euphorbiaceae	Leaves, seed	Ethanol	DPPH (l,l-diphenyl-2-picrylhydrazyl), Gallic acid, quercetin, gentisic acid, rutin, epicatechin and ellagic acid	Emetic, narcotic, purgative, swelling, prolapse of uterus, gastro-intestinal diseases, rheumatism, paralysis, asthma, cough and constipation	Seed oil mixed with decoction of jaman (*Cordia Oblique*) leaves is given to cattle for constipation problems and increase appetite. Its leaf extract with (Grewia sp) bark fiber and fruit is frequently used for prolapse of uterus and easy delivery and to hasten release of birth in cattle.	Oral, dermal	[34],[40],[71]
*Solanum surrattense* Burrn. f. Solanaceae	Whole plant	Methanol		Chest pain, vomiting, burning feet, cough, asthma, expectorant, stomachache, diuretic, gonorrhea, urinary, gastro-intestinal diseases	Fruit is dried, crushed and powder is taken for abdomen pain and gas trouble.	Oral	[29],[36],[59]
*Solanum xanthocarpum* Schrad. and Wendl. Sert. Hanov. Solanaceae	Leaves, stem, flower, root	Ethanol	Carpesterol and four steroidal glycosides, alkaloids, sterols, saponine, flavonoids, glycosides				[72],[73]
*Trigonella foenum graecum* L. Leguminosae	Seeds, stem, Leaves	Ethanol	5,7,3′-trihydroxy-5′-methoxylisoflavone, biochanin A, formononetin, irilone, tricin, daidzein,calycosin, orientin-2″-O-р-trans-coumarate, vitexin-2″-O-p-trans-coumarate, and tricin-7-O-β-D-glucopyranoside	anticancer, anti-inflammatory, antiseptic, aphrodisiac, astringent, anthelmintic, wound healing, gastroprotective, chronic cough, leprosy, heart disease, antidiabetic, diarrhea, urethera prolapse	200 g seeds are ground and the resulting powder is used orally after washing urethra with a sugar and potash alum (potassium alum) mixture for 4–5 days.50 g seeds are mixed with fodder and fed to animal for 3–4 days.	Oral	[34],[74],[75]
*Viscum album* L. Loranthaceace	Leaves, twigs	Ethyl acetate, chloroform, ethanolic, methanolic, aqueous	4′-O-[β-D-Apiosyl (1 → 2)]-β-D-glucosyl]-5-hydroxyl-7-O-sinapylflavanone, 3-(4-acetoxy-3,5-dimethoxy)-phenyl-2E-propenyl-β-D-glucopyranoside, 3-(4-hydroxy-3,5-dimethoxy)-phenyl-2E-propenyl-β-D-glucopyranoside, 5,7-dimethoxy-4′-O-β-D-glucopyranoside flavanone, 4′,5-dimethoxy-7-hydroxy flavanone, and 5,7-dimethoxy-4′-hydroxy flavanone	Anti-inflammatory, emetic, purgative, anti – diabetic, hernia			[76]
*Withania somnifera* Dunal. Solanaceae	Fruit, leaves	Methanol, ethanolic	withanosides I, II, III, IV, V, VI, and VII	Anthelmintic, leucorrhoea, tuberculosis abdominal pain	Fruit is given to children for removing abdominal pain. Decoction is used for blood purification.	Oral	[35],[31]
*Ziziphus vulgaris* Miller*.* Rhamnaceae	Fruit	Methanol	3-O-robinobioside, quercetin 3-O-rutinoside, 3-O-α-L-arabinosyl-(1 → 2)-α-L-rhamnoside, 3-O-β-D-xylosyl-(1 → 2)-α-L-rhamnoside, 3′,5′-di-C-β-D-glucosylphloretin, 3-O-β-D-xylosyl-(1 → 2)-α-L-rhamnoside-4′-O-α-L-rhamnoside,	Laxative, cutaneous and gastro-intestinal diseases	Infusion	Oral	[29],[35]

**Table 2:** **Antibacterial activities of Pakistani medicinal plants against** ***E. coli*** **at different concentration**

**Plant Species**	**Location**	**Part used**	**Extract**	**Concentration (mg/ml)**	**Zone of inhibition (mm)**	**References**
*A. officinalis*	Muzaffarabad	Root, leaves, flower	Methanol	15 (D)	1.9	[77]
*A. indica*	Faisalabad	Leaves	Ethanolic	50 (C)	18	[78]
				75 (C)	23	
*C. arvensis*	Cherat, Mardan, Malakand, Kohat	Leaves	Ethanolic	10 (C)	18	[30]
*C. procera*	Kohat	Stem	n-hexane	4 (D)	4	[17]
			Methanol	4 (D)	5.1	
			Aqueous	4 (D)	5	
			Chloroform	4 (D)	5.5	
			Butanol	4 (D)	6	
			n-hexane	2 (D)	3	
			Methanol	2 (D)	3	
			Aqueous	2 (D)	3.4	
			Chloroform	2 (D)	2	
			Butanol	2 (D)	3.5	
		Leaves	n-hexane	4 (D)	8.1	
			Aqueous	4 (D)	7.9	
			chloroform	4 (D)	7.9	
			Butanol	4 (D)	6	
			n-hexane	2 (D)	6.8	
			Aqueous	2 (D)	6	
			chloroform	2 (D)	5.2	
			Butanol	2 (D)	5.1	
*C. sativa*	Cherat, Mardan, Malakand, Kohat	Leaves	Ethanolic	10 (C)	20	[30]
*C. copticum*	Kohat		Methanol	50 (D)	10	[9]
			Ethanol	50 (D)	11	
			n-hexane	50 (D)	8	
*C. Intybus*	Sawabi, Gawadar		n-hexane	20 (D)	6.3	[79]
			Chloroform	20 (D)	7	
			Ethyl acetate	20 (D)	6.3	
*C. Intybus*	Mardan	Roots	Methanol	20 (D)	7.4	[80]
			n-hexane	20 (D)	5	
			chloroform	20 (D)	6.2	
			Ethyl acetate	20 (D)	7	
*Cichorium Noenum* L. Asteraceae	Sawabi, Gawadar		Methanol	20 (D)	3.4	[79]
			n-hexane	20 (D)	7	
			Chloroform	20 (D)	5	
			Ethyl acetate	20 (D)	5	
			Aqueous	20 (D)	3. 4	
*C. zeylanicum*	Kohat		n-hexane	50 (D)	10	[9]
*C. tubulosa*	KDA Karak		Methanolic	4 (D)	6	[81]
				2 (D)	4.1	
			Aqueous	4 (D)	4	
				2 (D)	3	
				1 (D)	1	
			Ethyl acetate	4 (D)	5.5	
				2 (D)	3.1	
			Chloroform	4 (D)	4.5	
				2 (D)	4	
			n-hexane	4 (D)	5	
				2 (D)	4.1	
				1 (D)	3.2	
			n-botanol	4 (D)	7	
				2 (D)	6.5	
				1 (D)	5	
*C. arvensis*	Peshawar	Leaves	Methanol	0.1 (C)	5	[82]
			n-hexane	0.1 (C)	6.2	
			Chloroform	0.1 (C)	7.1	
			Ethyl acetate	0.1 (C)	6.2	
		Stem	Methanol	0.1 (C)	4	
			n-hexane	0.1 (C)	6	
			Chloroform	0.1 (C)	6.2	
			Ethyl acetate	0.1 (C)	5.5	
			Aqueous	0.1 (C)	3	
		Root	Methanol	0.1 (C)	3.2	
			n-hexane	0.1 (C)	3.7	
			Chloroform	0.1 (C)	5.3	
			Ethyl acetate	0.1 (C)	3.3	
*D. innoxia*	Mirpur (Azad Jammu Kashmir)		Methanol	3 (C)	2	[8]
				5 (C)	3.5	
				7.5 (C)	4.5	
				10 (C)	6	
				12.5 (C)	7	
				15 (C)	8	
*D. regia*	Karachi	Flower	Ethanol	100 (C)	10	[12]
*D. viscosa*	Kohat	Aerial parts	Ethanolic	3.2 (D)	11	[11]
*E. gerardiana*	Baluchistan	Whole plant	Methanol	15 (D)	2.6	[77]
				12.5 (D)	1.6	
				10 (D)	1.5	
				7.5 (D)	1.5	
				5 (D)	1.4	
*E. camaldulensis*	Cherat, Mardan, Malakand, Kohat	Leaves	Ethanolic	10 (C)	18	[30]
*F. carica*	Cherat, Mardan, Malakand, Kohat	Leaves	Ethanolic	10 (C)	23	[30]
*G. glabra*	Peshawar	Root	Methanol	15 (D)	3.6	[77]
				12.5 (D)	1.6	
				10 (D)	1.5	
				7.5 (D)	1.4	
				5 (D)	1.4	
*H. officinalis*	Azad Jammu Kashmir	Leaves	Methanol	15 (D)	1.4	[77]
				12.5 (D)	1.2	
*J. adhatoda*	Margalla Hills	Leaves	Methanol	15 (D)	2	[77]
				12.5 (D)	1.6	
				10 (D)	1.5	
				7.5 (D)	1.4	
				5 (D)	1.4	
*M. neglecta*	Swat		Methanolic	4 (D)	13	[83]
			n-hexane	4 (D)	13.5	
			Chloroform	4 (D)	14	
			Aqueous	4 (D)	10	
*M. sylvestris*	Rawal Dam	Leaves, root, flower	Methanol	15 (D)	1.8	[77]
				12.5 (D)	1.6	
				10 (D)	1.6	
				7.5 (D)	1.6	
				5 (D)	1.4	
*M. longifolia*	Cherat, Mardan, Malakand, Kohat	Leaves	Ethanolic	10 (C)	18	[30]
*N. microphyllum.*	Swat		n-hexane	4 (D)	13	[83]
			Chloroform	4 (D)	12	
			Aqueous	4 (D)	10	
*O. europaea*	Cherat, Mardan, Malakand, Kohat	Leaves	Ethanolic	10 (C)	18	[30]
*O. limbata*	Cherat, Mardan, Malakand, Kohat	Leaves	Ethanolic	10 (C)	13	[30]
*Otostegia limbata*	Abottabad	Aerial parts	Ethanolic	8 (C)	11.5	[1]
			methanolic	8 (C)	6	
*Phyllanths emblica*	Kohat		Methanol	50 (D)	9.66	[9]
*R. communis*	Karachi	Leaves	Ethanol	100 (C)	12	[12]
*S. surrattense*	Mirpur (Azad Jammu Kashmir)		Methanol	3 (C)	1	[8]
				5 (C)	2.5	
				7.5 (C)	3	
				10 (C)	3.8	
				12.5 (C)	4	
				15 (C)	5	
*S. xanthocarpum*	Lahore	Leaves, stem, flower, root	Ethanolic	5 (C)	11.84	[84]
				50 (C)	14.52	
				100 (C)	16.78	
*T. foenum*	Karachi	Seeds	Ethanol	100 (C)	10	[12]
*V. album*	Azad Jammu Kashmir	Leaves, twigs	Ethyle acetate	100 (C)	24.96	[85]
			Chloroform	100 (C)	19.76	
			Ethanolic	100 (C)	16.66	
			Methanolic	100 (C)	16.93	
			Aqueous	100 (C)	9.16	
*W. somnifera*	Mirpur (Azad Jammu Kashmir)		Methanol	3 (C)	1	[8]
				5 (C)	3.7	
				7.5 (C)	4	
				10 (C)	7	
				12.5 (C)	8	
				15 (C)	8	
*W. somnifera*	Cherat, Mardan, Malakand, Kohat	Leaves	Ethanolic	10 (C)	18	[30]
*Z. vulgaris*	Mianwali	Fruits	Methanol	15 (D)	1.4	[77]

C = Concentration in the respective solvent; D = Concentration in DMSO solvent.

#### Justicia adhatoda

*J. adhatoda* is traditionally being used for the treatment of variety of diseases caused by *E. coli* and other microorganisms (Table 1). Limited inhibition of methanolic extracts of its leaves was found against *E. coli* (Table 2). *J. adhatoda* showed 2 mm inhibition at 15 mg/ml concentration, 1.6 mm inhibition at 12.5 mg/ml concentration, 1.5 mm at 10 mg/ml and 1.4 mm at 7.5 mg/ml concentration when dissolved in DMSO (Table 2). Studies have revealed the presence of alkaloids in the methanolic extract of its leaves (Table 1). Alkaloids isolated from *J. adhatoda* showed bronchodilator activity [86], however not evaluated for antimicrobial activities.

#### Althaea officinalis

Roots of *A. officinalis* are very useful and traditionally used for intestinal and respiratory problems. The methanolic extract of roots, leaves and flowers of *A. officinalis* when dissolved in DMSO have shown 1.9 mm inhibition against *E. coli* (Table 2). Phytoconstituents like altheahexacosanyl lactone (Figure 4.1), altheacalamene, β-sitositerol, altheacoumarin glucoside and other constituents have been obtained from the methanolic and ethanolic extract of root, seeds and leave of *A. officinalis* (Table 1). The anti-*E. coli* activity of this plant could be due to presence of these compounds. However, further studies are required in order to find out the constituents that may have strong potential against *E. coli.*

#### Azadirachta indica

Ethnomedicinally, *A. indica* is considered one of medicinal plants having great potential against variety of diseases (Table 1). For instance, the decoction of leaves is used for curing digestive and gastric problems. Leaves are dried, crushed and powder is mixed with small quantity of water and taken as remedies of freckles on face and increase appetite by lessening stomach flatulence and killing intestinal worms [35],[36]. Ethanolic extract of leaves of *A. indica* showed 18 and 23 mm inhibition at 50 and 75 mg/ml, respectively against *E. coli*[78]. More than 135 compounds have been isolated so far from different parts of *A. indica,* however not of them are studied for their biological activities. Nimbin (Figure 4.2) and Nimbidin are major crude bitter principle extracted from the oil of seed kernels of *A. indica*, which have demonstrated several biological activities including antifungal, antibacterial and anti-inflammatory [76].

#### Mentha longifolia

Traditionally the decoction of *M. longifolia* is used for cholera, diarrhea and stomach problems in the rural area of Pakistan [61]. The ethanolic extract of leaves of *M. longifolia* showed 18 mm inhibition against *E. coli* when dissolved in their respective solvent at 10 mg/ml (Table 1). Monoterpenes and sesquiterpenes present in aerial parts were found to possess antibacterial activities [87]. *M. longifolia* has also been scientifically proved for its insecticidal [87], antispasmodic and antiplatelet properties [88].

#### Delonix regia

Ethnomedicinally, the root of *D. regia* has been proved very potent against abdominal pain while leaves are used as anti-inflammation. *In-vitro* ethanolic extract of *D. regia* has shown 10 mm zone of inhibition at 100 mg/ml concentration against *E. coli*[12] (Table 2). Large number of phytoconstituents such as tannins, triterpenoids, steroids, glycosides, flavonoids, L-Azetidine-2-carboxylic acid, lupeol etc. have been isolated from the plant when extracted with alcoholic solvents (Table 1).

#### Dodonaea viscosa

Traditional healers of Pakistan use mostly leaves’ infusion of *D. viscose* for curing different diseases caused by microbial agents (Table 1). The ethanolic extract of aerial parts of *D. viscose* dissolved in DMSO solvent at 3.2 mg/ml concentration showed 11 mm inhibition zone [11] against *E. coli* (Table 2) that might be associated with the presence of tannins, saponins, flavonoids and terpenoids in the studied parts [51].

### Medicinal plants extracted with butanol

Butanolic extracts of Pakistani medicinal plants also showed optimum anti-*E. coli* inhibitory activities. However, the use of butanol for plant extraction is very limited in Pakistan. Present review reported only 2 plants out of 34 extracted with butanol (Figure 1). Increase in concentration of butanolic extract in DMSO has significantly increased the inhibition zone against *E. coli* (Figure 3).

#### Calotropis procera

Traditional healers in the remote areas of Pakistan use *C. procera* in the form of infusion against gastro-intestinal troubles. The butanolic extract of stem and leaves of *C. procera* showed 6 mm inhibition against *E. coli* at 4 mg/ml concentration dissolved in DMSO (Table 2). Saponins, alkaloids, triterpenoids and flavonoids classes of compounds might be responsible for its strong anti-*E. coli* activity [39].

#### Cistanche tubulosa

No traditional uses of this plant has been reported in Pakistan, however, *in-vitro* scientific validation against *E. coli* provide a strong base for this plant to be used as potent medicinal plant. Butanolic extract of *C. tubulosa* dissolved in DMSO showed 7 and 6.5 mm inhibition against *E. coli* at concentration of 4 and 2 mg/ml, respectively (Table 2). Secondary metabolites such as glycosides and monoterpenes have been isolated from the butanolic extracts of this plant.

### Medicinal plants’ aqueous extracts

Water is also used as a solvent for the extraction of medicinal plants in Pakistan. Extraction of plants with organic solvents gives more consistent antimicrobial activities as compared to inorganic solvents [89]. Reason behind less activities of inorganic solvent might be due to the presence of better medium for growth and occurrence of microorganisms [90]. Furthermore, water-soluble compounds, such as polysaccharides and polypeptides have no real impact as antimicrobial agents [16]. This could be the main reason of limited use of water for the extraction of medicinal plants in Pakistan. Present review showed that only 2 plants that have been extracted using water as solvent (Figure 1). Negative correlation was found between concentration of aqueous extract of medicinal plants and the zone of anti-*E. coli* inhibition (Figure 3).

#### Calotropis procera

*C. procera* has shown antibacterial activities due to the presence of different phytoconstituents like flavonoids, tannins etc. Aqueous extracts of the leaves of *C. procera* showed 7.9 mm (4 mg/ml) and 6 mm (2 mg/ml) inhibition against *E. coli*, when dissolved in DMSO [17].

#### Malva neglecta

Traditionally the plant is used against gastro-intestinal problems [40]. Aqueous extract of *M. neglacta* showed anti-*E. coli* inhibition zone of 10 mm on dissolving in DMSO at 4 mg/ml concentration [84] (Table 2). No study has been reported on the phytochemical screening of *M. neglacta* (Table 1).

### Medicinal plants extracted with ethyl acetate

Literature review has shown limited use of ethyl acetate for the extraction of medicinal plant in Pakistan against *E. coli*. Out of 34 medicinal plants, only 2 were extracted with ethyl acetate that showed inhibition against *E. coli* (Figure 1). Significant positive correlation was observed between the concentration of pure ethyl acetate extract and anti-*E. coli* inhibitory potential (Figure 2). Increase concentration of ethyl acetate in DMSO also increases inhibition potential again *E. coli,* however statistically it not significant (Figure 3).

#### Viscum album

Traditionally, local people use *V. album* for variety of ailments like gastro-intestinal and anti-inflammatory (Table 1). The ethyl acetate extract of *V. album* showed 24.96 mm inhibition against *E. coli* at concentration of 100 mg/ml that might be due to the presence of variety of active phytoconstituents like 4′-O-[β-D-Apiosyl (1 → 2)]-β-D-glucosyl]-5-hydroxyl-7-O-sinapylflavanone, 3-(4-acetoxy-3,5-dimethoxy)-phenyl-2E-propenyl-β-D glucopyranoside and 5,7-dimethoxy-4′-hydroxy flavanone etc. [91],[92].

#### Cichorium intybus

*C. intybus* is traditionally used for the treatment of abdominal pain and diarrhea (Table 1). Root extract of *C. intybus* showed 7 mm inhibition against *E. coli* at 20 mg/ml concentration dissolved in DMSO solvent [80]. Antibacterial activity of *C. intybus* might be associated with the presence of different phytoconstituents (Table 2). There is no study reported on the ethyl acetate soluble phytoconstituents in the world. However, methanol soluble phytochemical are [lup-12,20 (29)-dien-3β-ol-3β-L-arabinofuranosyl-2′-hexadecanoate],[lup-12,20 (29)-dien-3β-olyl hexadecanoate] and [4β-(pent-2-enylolactone)-hexatriacontane] (Table 1).

### Medicinal plants extracted with chloroform

Literature study has indicated chloroform with high inhibition against *E. coli*. In total, 7 medicinal plants were extracted with chloroform (Figure 1). Significant positive correlation was found between anti-*E. coli* inhibition zone and concentration of plant extracts in their respective solvent.

#### Malva neglecta

Ethnomedicinal properties of *M. neglacta* have already been discussed above. Chloroform extract of *M. neglacta* showed 14 mm zone of inhibition against *E. coli* at 4 mg/ml when dissolved in DMSO [83] (Table 2).

#### Cichorium intybus

Chloroform extract of *C. intybus* has shown *in-vitro* inhibitory activity against *E. coli.* It showed 6.22 mm anti-*E. coli* inhibition zone at concentration of 20 mg/ml dissolved in DMSO [80].

### Medicinal plants extracted with n-hexane

N-hexane extracts does not exhibit better anti-*E. coli* activities. N-hexane extracts of different plants like *Terminalia catappa* and *Dodonaea viscose* have been found with no antibacterial activities [11],[93]. However, the present review showed that certain plants exhibit antibacterial activities when extracted with n-hexane solvent. In total, 10 out of 34 medicinal plants were extracted with n-hexane showing antibacterial activity (Figure 1), which could be due to difference in the phytochemistry between plants [27].

#### Cinnamomum zeylanicum

Traditional importance of *C. zeylanicum* could be observed due to its varied utilization against different ailments (Table 1). N-hexane extract of *C. zeylanicum* at 50 mg/ml in DMSO has shown 10 mm inhibition against *E. coli*[9] (Table 2) that might be due to the presence of active phytoconstituents isolated from the bark of *C. zeylanicum*[45].

#### Carum copticum

Ethnomedicinally, *C. copticum* induces appetite, remove kidney stone as well use for the treatment of digestion and whooping cough. Its seeds are taken with salt for gastric trouble (Table 1). According to Shinwari *et al.*[9] the n-hexane extract of this plant showed 8 mm zone of inhibition against the *E. coli* at 50 mg/ml in DMSO (Table 2). There is no study conducted so far on the phytochemistry of this plant.

## Conclusions

The present review concluded that inhabitants of remote areas of Pakistan are greatly dependent on ethnomedicinal plants for the treatment of different ailments caused by *E. coli*. Majority of medicinal plants have been proved *in-vitro* for their therapeutic activities against *E. coli*. Different organic and inorganic solvents have been used in Pakistan for medicinal plants extraction, however, methanol being used the most. Different compounds such as nimbin, alkaloids of *J. adathoda*; glycosides of *W. somnifera* etc. were found inhibiting the growth of *E. coli*. Ethnomedicinal knowledge provides baseline information for the search of novel drugs and compounds against variety of infectious diseases cause by microorganisms. Therefore, detailed ethnomedicinal studies should be carried out in Pakistan in order to conserve this valuable knowledge before its extinction. Moreover, solvents other than methanol should also give preference in future as it could lead to the separation of some new therapeutic compounds that could be active against *E. coli*. Phytochemical screening of unexplored plants like *M. neglecta*, *C. copticum* etc. should be given focussed as it could result in development of new antimicrobial drugs with fewer side effects.

## Competing interests

The authors declare that they have no competing interests.

## Authors’ contributions

All authors have fully contributed in writing and revising the manuscript critically. All authors read and approved the final manuscript.
